# Treating Refractory Cardiogenic Shock With the TandemHeart and Impella Devices: A Single Center Experience

**DOI:** 10.4021/cr121w

**Published:** 2012-03-20

**Authors:** Bryan G. Schwartz, Daniel J. Ludeman, Guy S. Mayeda, Robert A. Kloner, Christina Economides, Steven Burstein

**Affiliations:** a Heart Institute, Good Samaritan Hospital, Los Angeles, California, USA; b Department of Cardiology, Good Samaritan Hospital, Los Angeles, California, USA; c Department of Internal Medicine, Division of Cardiovascular Medicine, Keck School of Medicine at the University of Southern California, Los Angeles, CA, USA

**Keywords:** Cardiopulmonary resuscitation, Heart-assist devices, Heart failure, Hemodynamics, Myocardial infarction, Cardiogenic shock

## Abstract

**Background:**

Patients with cardiogenic shock (CS) are routinely treated with intra-aortic balloon pumps (IABPs). The utility of 2 new percutaneous left ventricular assist devices (PLVADs), the Impella and TandemHeart, is unknown. The objective of this study was to describe the use of PLVADs for patients with CS at our institution.

**Methods:**

All cases involving PLVADs in patients with CS between between January 1, 2008 and June 30, 2010 at a private, tertiary referral hospital were reviewed retrospectively.

**Results:**

All 76 cases were identified (50 IABP only, 7 Impella, 19 TandemHeart). Most Impella (5/7) and TandemHeart (10/19) patients were initially treated with an IABP before "upgrading" for increased hemodynamic support. All 76 devices (100%) were initiated successfully. Percutaneous revascularization was attempted in 63 patients with angiographic success in 57 (90%). The incidences of major complications were similar between groups, except bleeding occurred less frequently with the IABP. Mean ejection fraction on presentation was 30.4±16.5% and increased by a mean of 6.6±11.4% (P < 0.001). With the institutional approach of treating patients with CS initially with vasopressors and IABPs, then upgrading to an Impella or TandemHeart device for patients refractory to IABP therapy, the overall mortality rate was 40%.

**Conclusion:**

The Impella and TandemHeart devices can be initiated successfully in patients with CS, are associated with high rates of angiographic success during high risk percutaneous interventions and may benefit the myocardium during myocardial infarction. Randomized trials are warranted investigating use of the Impella and TandemHeart devices in patients with CS and in patients refractory to conventional IABP therapy.

## Introduction

Cardiogenic shock (CS) is associated with a mortality of approximately 60-70% [1-3]. With early revascularization and intra-aortic balloon pump (IABP) therapy, mortality occurs in approximately 45-50% of patients with CS [1-3]. Although no randomized trial has demonstrated a mortality benefit, patients with CS are routinely supported hemodynamically with vasopressors and IABPs [4], and use of IABPs for patients with CS is supported by recent guidelines [5]. In recent years, 2 new percutaneous left ventricular assist devices (PLVADs) were introduced, the TandemHeart (CardiacAssist, Inc., Pittsburgh, PA) and Impella Recover 2.5 (Abiomed Inc., Danvers, MA, USA) devices. In 3 small, randomized trials of patients in CS the new PLVADs provided superior hemodynamic support compared with the IABP; however, they also increased certain complication rates, including bleeding and leg ischemia [6-9]. A mortality benefit was not found in these 3 randomized trials comparing the new PLVADs with the IABP, but a total sample size of 100 patients limited the ability to identify any potential significant difference and data was not available to conduct a subanalysis to potentially identify subgroups of patients that may benefit from the new PLVADs [7]. Clinical experience with these new devices is limited to 3 small randomized trials [6-9] and small retrospective analyses [10-15]. Compared with the IABP, the TandemHear and Impella devices may be more beneficial for patients in CS with severely depressed cardiac function as they provide increased hemodynamic support; however, their role in clinical practice is currently unknown. Thus, the purpose of this study is to investigate the baseline characteristics, hemodynamic parameters and outcomes of patients in CS treated with the currently available PLVADs to better define their use in contemporary practice.

## Methods

Approval was obtained from the Western Institutional Review Board for this retrospective analysis at a private, tertiary referral hospital. The current report represents an expansion of a previous description [12] of patients treated with a TandemHeart device only. The current study includes additional patients treated with a TandemHeart, as well as patients treated with an IABP or Impella, and includes additional endpoints that were not explored previously. All cases involving IABPs or PLVADs (Impella, or TandemHeart) were identified by searching the electronic medical record database between January 1, 2008 and June 30, 2010. Each device was available during the entire study period. All cases were included in which an IABP or PLVAD was used for hemodynamic support in a patient with CS. The use of each device was further defined as emergent if the device was inserted emergently for hemodynamic support or elective if the device was placed electively to facilitate a high risk percutaneous coronary intervention in a patient suffering from CS who had already stabilized with vasopressors ± an IABP. All clinical decisions were at the discretion of the treating physicians, including the decision to use a PLVAD, the type of device and the type of PCI. Demographics, medical history, procedural characteristics, in-hospital outcomes and outcomes at 30 days (if available) were recorded through a comprehensive chart review.

### Device selection

The general approach at this institution was to treat patients in CS initially with an IABP, especially in an emergent setting because IABPs are relatively simple and rapid to initiate. Moreover, most cardiac care unit personnel are experienced with IABPs and there is extensive literature on IABPs. Therefore, most patients in the IABP group presented emergently with an ST-segment elevation myocardial infarction (STEMI) (70%) or cardiac arrest (16%). Then, if the IABP was deemed inadequate, hemodynamic support was increased with implementation of an Impella or a TandemHeart device. Thus, many of the Impella (5 of 7) and TandemHeart (10 of 19) patients had failed therapy with vasopressors and an IABP. The decision between an Impella or TandemHeart device was based primarily on hemodynamics such that the TandemHeart device was reserved for patients with the most severe hemodynamic compromise. This approach reflects the greater degree of support provided by the TandemHeart (3.5 - 5 L/min with a 15 - 17 Fr inflow cannula) compared with the Impella (2.5 L/min) and because the Impella requires some inherent left ventricular function and blood flow to operate whereas the TandemHeart provides output independent of left ventricular function. Accordingly, immediately prior to device initiation, patients in the Impella group had a mean (vasopressor supported) systolic blood pressure of 105 mmHg with a mean of 1.3 vasopressors, compared to the TandemHeart group which had a mean (vaspressor supported) systolic blood pressure of 93 mmHg with a mean of 1.7 vasopressors. In addition, two patients had an undetectable blood pressure prior to TandemHeart initiation. Furthermore, an ejection fraction ≤ 25% was more prevalent in the TandemHeart patients (71%) than the Impella patients (57%). The TandemHeart was also used once each in a patient with myocarditis and with severe mitral regurgitation.

### IABP insertion technique

An 8 Fr, 40 cc Fidelity IAB IABP (MAQUET Cardiovascular, Fairfield, NJ) was inserted via either femoral artery using the retrograde guidewire technique and was placed inferior to the left subclavian artery.

### Impella insertion technique

Two Perclose devices (Abbott Vascular, Redwood City, CA) were used to "preclose" the femoral artery, then a 13 Fr sheath was inserted. Through a pigtail catheter, a 0.038 inch guidewire was passed across the aortic valve. The guidewire was exchanged for a dedicated 0.018 inch Impella guidewire and the pigtail catheter was removed. Next, the Impella device was advanced retrogradely across the aortic valve and into the left ventricle. Fluoroscopy was used to confirm proper positioning. Finally, the Impella device was activated at the lowest level (P1) and the setting was titrated to achieve approximately 2.5 L/min of hemodynamic support.

### TandemHeart insertion technique

Via the right femoral vein, transeptal puncture was performed using a Brockenbrough needle and a modified Mullins sheath. Next, a stiff 0.035 inch guidewire was placed in the left atrium. The Mullins sheath was exchanged for a 14/21 two-stage dilator, which was then exchanged for the 21 Fr TandemHeart transeptal cannula. The external end of the TandemHeart cannula was secured to the patient’s right thigh. In nonemergent cases, the left femoral artery was "preclosed" then upsized to a 15 or 17 Fr TandemHeart arterial cannula, which was placed superior to the aortic bifurcation and its external end was secured to the patient’s left thigh. After removing air from the system, the venous and arterial cannulae were connected to the external TandemHeart centrifugal pump. The pump was cooled, lubricated and anticoagulated with heparinized saline, and attached to the control system. Pump speed was titrated to achieve adequate hemodynamic support (often 3.5 to 5 L/min).

### Definitions

Clinical presentation was defined as STEMI if there was ST-segment elevation > 1 mm in contiguous leads (> 2 mm in precordial leads) or non-STEMI if there was an elevation in troponin I > 1.0 ng/mL (laboratory standard) or an elevation in creatine kinase > 2 times the upper limit of normal with a rise in creatine kinase-MB. Cardiogenic shock was defined as persistent systolic blood pressure < 90 mmHg, or if vasopressor or PLVAD support was necessary to maintain a systolic blood pressure > 90 mmHg. Additional hemodynamic criteria and evidence of end-organ hypoperfusion were not required for this retrospective analysis; however, patients were excluded if hemodynamic parameters indicated shock from a noncardiac cause. Cardiopulmonary resuscitation (CPR) was defined as pulseless arrhythmias requiring chest compressions and/or cardiac defibrillation or emergent intubation for respiratory failure. Angiographic success was defined as TIMI grade 3 flow and < 30% residual stenosis at the conclusion of the procedure; or, for cases of aortic valvuloplasty, a final gradient across the aortic valve of < 25 mmHg or a ≥ 50% reduction in aortic valve gradient. Procedural success was defined as angiographic success without death while in the catheterization laboratory. TIMI minor and TIMI major bleeding were defined as a decrease in hemoglobin of 3.0 - 4.9 g/dL and ≥ 5.0 g/dL, respectively (each unit of transfused packed red blood cells was counted as a decrease in hemoglobin of 1.0 g/dL).

For hemodynamic data, "before" was defined as the most recent value prior to device insertion, "during" was defined as 30 minutes after device insertion, and "after" was defined as 30 - 120 minutes after the device removal. For hemodynamic data, the overall data trend and surrounding data points were evaluated and outlier values were avoided. For platelets and hemoglobin, the "before" value was the most recent value prior to device insertion, and the "after" value was defined as the lowest value during device use or within 3 days of device discontinuation. Ejection fraction was determined prospectively, by visual estimation, without regard to this research project. Ejection fraction was obtained preferably by echocardiography. Left ventriculogram was used in patients who presented emergently, precluding initial echocardiography evaluation. The "after" ejection fraction was defined as the first documented ejection fraction after device removal, which generally occurred within 7 days. Before-during change was calculated by subtracting the before value from the during value. Before-after change was defined as the after value minus the before value.

### Statistics

Results are reported as the mean ± standard deviation or percentages of the total. Cases were stratified into 3 groups based on PLVAD used: IABP, Impella, or TandemHeart. Direct comparisons were not made between groups because selection bias resulted in different degrees of hemodynamic compromise at baseline (IABP the least, Impella intermediate, and TandemHeart the most compromised). To test for possible differences in ejection fraction and systolic blood pressure measurements before and after the device was used, a paired t-test for means was done using Microsoft Office Excell. Statistical significance was considered a p-value < 0.05.

## Results

A total of 138 cases were identified, which included 76 patients in cardiogenic shock. Fifty patients were treated only with an IABP, 7 with an Impella and 19 with a TandemHeart (Table 1). Most Impella patients (5/7) and TandemHeart patients (10/19) were initially treated with an IABP before "upgrading" to a PLVAD for increased hemodynamic support. Mean age was 67.9 ± 11.9 years and patients were mostly male (65%). Hypertension, diabetes, hyperlipidemia and renal insufficiency were each present in at least 50% of patients. Mean ejection fraction was 30.4±16.5%. An ejection fraction ≤ 25% was present in 41% of all patients and was more prevalent in the Impella (57%) and TandemHeart patients (71%).

**Table 1:** Patient Demographics and Medical History

	Total	IABP	IMP	TH
Number of Patients	76	66% (50/76)	9% (7/76)	25% (19/76)
IABP Prior to PLVAD			71% (5/7)	53% (10/19)
IABP During PLVAD			57% (4/7)	21% (4/19)
IABP After PLVAD			14% (1/7)	5% (1/19)
Age (years)	67.9 ± 11.9	67.4 ± 11.4	69.7 ± 10.5	68.5 ± 14.1
Male Gender	65% (49/76)	58% (29/50)	86% (6/7)	74% (14/19)
Race, Caucasian	13% (10/76)	12% (6/50)	14% (1/7)	16% (3/19)
Asian	26% (20/76)	24% (12/50)	14% (1/7)	37% (7/19)
Hispanic	41% (31/76)	42% (21/50)	43% (3/7)	37% (7/19)
Black	11% (8/76)	12% (6/50)	0% (0/7)	11% (2/19)
Unknown/Other	9% (7/76)	10% (5/50)	29% (2/7)	0% (0/19)
Hypertension	68% (52/76)	68% (34/50)	57% (4/7)	74% (14/19)
Diabetes Mellitus	55% (42/76)	64% (32/50)	57% (4/7)	32% (6/19)
Insulin-Dependent	17% (13/76)	24% (12/50)	0% (0/7)	5% (1/19)
Hyperlipidemia	51% (39/76)	56% (28/50)	71% (5/7)	32% (6/19)
Atrial Fibrillation	7% (5/76)	8% (4/50)	0% (0/7)	5% (1/19)
Current Smoker	22% (17/76)	18% (9/50)	29% (2/7)	32% (6/19)
Remote Smoker	21% (16/76)	18% (9/50)	14% (1/7)	32% (6/19)
Remote PCI	20% (15/76)	22% (11/50)	57% (4/7)	0% (0/19)
Remote MI	13% (10/76)	12% (6/50)	43% (3/7)	5% (1/19)
Remote CABG	11% (8/76)	14% (7/50)	14% (1/7)	0% (0/19)
Creatinine (mg/dl)	1.9 ± 1.9 (75)	2.1 ± 2.2 (49)	1.5 ± 0.9 (7)	1.8 ± 1.2 (19)
% (n) ≥ 1.4	50% (38/75)	52% (26/50)	43% (3/7)	47% (9/19)
Hemodialysis	8% (6/76)	10% (5/50)	0% (0/7)	5% (1/19)
History Of Prior CHF	29% (22/76)	24% (12/50)	43% (3/7)	37% (7/19)
Ejection Fraction	30 ± 17 (62)	33 ± 16 (39)	22 ± 10 (6)	27 ± 18 (17)
% (n) ≤ 35%	55% (42/62)	56% (22/39)	100% (6/6)	82% (14/17)
% (n) ≤ 25%	41% (31/62)	39% (15/39)	57% (4/6)	71% (12/17)
Aortic Stenosis	9% (7/76)	8% (4/50)	0% (0/7)	16% (3/19)
Aortic Regurgitation	4% (3/76)	2% (1/50)	0% (0/7)	11% (2/19)
Mitral Regurgitation	12% (9/76)	14% (7/50)	14% (1/7)	5% (1/19)

IABP: Intra-Aortic Balloon Pump; IMP: Impella L.P. 2.5; TH: TandemHeart; PLVAD: Impella or TandemHeart

PCI: Percutaneous Coronary Intervention; MI: Myocardial Infarction; CABG: Coronary Artery Bypass Graft; CHF: Congestive Heart Failure

### Presentation

The most common presentations were STEMI and cardiac arrest, which were managed primarily with IABPs (Table 2). Six of 7 Impella patients presented with a myocardial infarction (3 STEMI, 3 non-STEMI). The TandemHeart device was utilized more for patients with severely decompensated congestive heart failure and once each for myocarditis and severe mitral regurgitation due to perivalvular leak. Emergent device placement occurred in 84% of all cases. The Impella and TandemHeart groups also included cases with elective device placement prior to high risk percutaneous intervention in patients suffering from CS after initial stabilization with vasopressors ± an IABP. Nearly all patients (99%) required vasopressor therapy prior to device initiation with a mean of 1.6 ± 0.6 agents, primarily dopamine.

**Table 2:** Presentation

	Total	IABP	IMP	TH
Transferred From OSH	40% (30/76)	34% (17/50)	57% (4/7)	47% (9/19)
Emergent	84% (64/76)	98% (49/50)	57% (4/7)	58% (11/19)
Elective	16% (12/76)	2% (1/50)	43% (3/7)	42% (8/19)
STEMI	59% (45/76)	70% (35/50)	43% (3/7)	37% (7/19)
Peri-operative STEMI	3% (2/76)	4% (2/50)	0% (0/7)	0% (0/19)
Cardiac Arrest	11% (8/76)	16% (8/50)	0% (0/7)	0% (0/19)
Non-STEMI	9% (7/76)	4% (2/50)	43% (3/7)	11% (2/19)
CHF	16% (12/76)	6% (3/50)	14% (1/7)	42% (8/19)
Other^a^	5% (4/76)	4% (2/50)	0% (0/7)	11% (2/19)
CS Began in Cath Lab	21% (16/76)	20% (10/50)	29% (2/7)	21% (4/19)
Received CPR	49% (37/76)	54% (27/50)	43% (3/7)	37% (7/19)
On Vasopressors	99% (75/76)	100% (50/50)	86% (6/7)	100% (19/19)
Mean Number of Vasopressors	1.6 ± 0.6	1.6 ± 0.6	1.3 ± 0.8	1.7 ± 0.7
Dopamine	83% (63/76)	82% (41/50)	71% (5/7)	90% (17/19)
Norepinephrine	50% (38/76)	54% (27/50)	43% (3/7)	42% (8/19)
Dobutamine	15% (11/76)	14% (7/50)	0% (0/7)	21% (4/19)
Epinephrine	5% (4/76)	2% (1/50)	0% (0/7)	16% (3/19)
Neosynephrine	7% (5/76)	6% (3/50)	14% (1/7)	5% (1/19)
Revascularization				
PCI	83% (63/76)	84% (42/50)	100% (7/7)	74% (14/19)
Failed Lytics (Rescue PCI)	11% (8/76)	8% (4/50)	29% (2/7)	11% (2/19)
Aortic Valvuloplasty	7% (5/76)	6% (3/50)	0% (0/7)	11% (2/19)
PCI and CABG	5% (4/76)	6% (3/50)	0% (0/7)	5% (1/19)
CABG Alone	4% (3/76)	4% (2/50)	0% (0/7)	5% (1/19)
None^b^	13% (10/76)	12% (6/50)	0% (0/7)	21% (4/19)
Type of Intervention				
PTCA	74% (56/76)	74% (37/50)	100% (7/7)	63%(12/19)
Thrombectomy	40% (30/76)	44% (22/50)	29% (2/7)	32%(6/19)
DES	40% (30/76)	34% (17/50)	71% (5/7)	42%(8/19)
BMS	21% (16/76)	22% (11/50)	14% (1/7)	21%(4/19)
RA	7% (5/76)	4% (2/50)	0% (0/7)	16%(3/19)
Aortic Valvuloplasty	7% (5/76)	6% (3/50)	0% (0/7)	11%(2/19)

a Includes 1 patient each with stable angina (IABP), post-ablation ventricular tachycardia (IABP), myocarditis (TH), severe mitral regurgitation (TH).

b includes patients with myocarditis, severe mitral regurgitation, pending CABG, pending transplant, early death. IABP: Intra-Aortic Balloon Pump; IMP: Impella L.P. 2.5; TH: TandemHeart; OSH: Out-Side Hospital; MI: Myocardial Infarction; CHF: Congestive Heart Failure; CS: Cardiogenic Shock; PCI: Percutaneous Coronary Intervention; CABG: Coronary Artery Bypass Graft; EF: Ejection Fraction; PTCA: Percutaneous Transluminal Coronary Angioplasty; DES: Drug Eluting Stent; RA: Rotational Atherectomy; BMS: Bare Metal Stent.

### Revascularization

Some form of revascularization or aortic valvuloplasty was performed in 83% of patients. In this cohort that included many STEMIs, balloon angioplasty (74%), thrombectomy (40%) and drug-eluting stent placement (40%) were the most common interventions performed. Of the 63 patients undergoing percutaneous interventions, angiographic success was achieved in 57 (90%), including 100% of the Impella and TandemHeart patients (Table 3).

**Table 3:** Procedural Complications and 30-Day Outcomes

	Total	IABP	IMP	TH
Successful Device Initiation	100% (76/76)	100% (50/50)	100% (7/7)	100% (19/19)
Angiographic Success	90% (57/63)	86% (36/42)	100% (7/7)	100% (14/14)
Procedural Success	89% (56/63)	86% (36/42)	100% (7/7)	93% (13/14)
Final Follow Up (Days)	12.5 ± 12.1	12.5 ± 11.9	18.4 ± 13.8	10.5 ± 11.7
30 Days	24% (18/76)	34% (17/50)	43% (3/7)	16% (3/19)
Death	40% (30/76)	34% (17/50)	14% (1/7)	63% (12/19)
Emergent Device Placement	41% (26/64)	35% (17/49)	25% (1/4)	73% (8/11)
Elective Device Placement	33% (4/12)	0% (0/1)	0% (0/3)	50% (4/8)
Did Not Receive CPR	28% (11/39)	17% (4/23)	0% (0/4)	58% (7/12)
Received CPR	51% (19/37)	48% (13/27)	33% (1/3)	71% (5/7)
Stroke	4% (3/76)	4% (2/50)	0% (0/7)	5% (1/19)
Limb Ischemia^a^	7% (5/76)	6% (3/50)	0% (0/7)	11% (2/19)
Other^b^	4% (3/76)	2% (1/50)	14% (1/7)	5% (1/19)

^a^3 resolved upon device removal, 1 resolved upon device adjustment, and none required surgery. ^b^Includes 1 each of acute arterial embolization of subclavian artery (IABP), clot seen on catheter in setting of apical aneurysm (Impella), and decrease in blood pressure to 60/40 during first attempt at transeptal puncture (TandemHeart, second attempt successful) IABP: Intra-Aortic Balloon Pump; IMP: Impella L.P. 2.5; TH: TandemHeart; CPR: cardiopulmonary resuscitation.

### Device success and device related complications

All 76 devices (100%) were initiated successfully and device related complications were minimal (Table 3). Mean duration of device use was 40.1 ± 36.4 hours and tended to be longer in the IABP group (48.7 ± 36.9 hours vs. 29.4 ± 28.5 with Impella vs. 21.4 ± 30.7 with TandemHeart). One TandemHeart insertion was complicated by a decrease in blood pressure to 60/40 mmHg during the initial attempt at transeptal puncture, possibly due to a vagal reaction. The patient required minimal cardiopulmonary resuscitation but responded rapidly and the second attempt at transeptal puncture was successful. A thrombus was seen on one Impella device on echocardiogram in the setting of an apical aneurysm. The device was removed without complications. One IABP was associated with acute subclavian artery embolization and cool, pulseless lower extremities. The IABP was removed, but the patient’s overall condition and lower extremities did not improve and the patient died. Four other patients (2 IABP, 2 TandemHeart) developed signs of limb ischemia that resolved upon device removal in 3 patients and upon device adjustment in the other. No patients required intervention for limb ischemia. Three patients suffered a stroke within 30 days, including 2 of 50 (4%) with an IABP and 1 of 19 (5%) with a TandemHeart device. The cause of stroke was not documented for 1 IABP patient; the other 2 were embolic strokes in patients with severe aortic atheromas (presumably unrelated to the TandemHeart device which did not come in contact with the aortic disease).

### Survival

Overall in-hospital death occurred in 30 of 76 (40%) patients (Table 3). Mean time from device insertion to death was 4.3 ± 5.8 days and 12 patients (16%) died within the first 24 hours. For patients in CS who underwent elective (not emergent) device placement prior to high risk percutaneous interventions the death rate was 33% (4 of 12). The death rate for the IABP group was only 34% (17 of 50) because 15 patients in whom the IABP was deemed inadequate were crossed over into the Impella and TandemHeart groups. Eight of these 15 patients (53%) died (1 of 5 Impella, 7 of 10 TandemHeart); it is unknown how many would have survived with IABP support alone. Overall, death occurred in 1 of 7 (14%) Impella patients and in 12 of 19 (63%) TandemHeart patients.

Thirty-seven patients (49%) received CPR prior to PLVAD placement (Table 3). The mortality rate was higher in patients who received CPR compared with patients who did not (51% vs. 28%).

### Bleeding Complications

Bleeding was a frequent complication with all 3 devices (Table 4). TIMI major bleeding occurred in 25 of 76 (33%) patients and tended to occur more frequently in the Impella and TandemHeart groups. TIMI minor bleeding occurred in 17 of 76 (22%) patients. There were no instances of retroperitoneal bleeding or bleeding that required surgical correction. Thirty-six of 76 patients (47%) received a transfusion of packed red blood cells (mean 4.1 ± 4.2 units). The TandemHeart patients tended to require more blood products. In this high risk cohort, 17 of 76 patients (22%) had a reason for bleeding unrelated to the IABP or PLVAD, including 6 of 19 patients (32%) in the TandemHeart group. Vascular closure devices were not used for IABPs and were used mainly for elective, not emergent placement of the Impella and TandemHeart devices. Bleeding complications may be reduced with the Perclose device (Abbott Vascular, Redwood City, CA) using the "preclose" technique, whereby the device is initiated first with lacing of the sutures through the vessel, then the PLVAD is deployed, and finally, after the PLVAD has been removed the Perclose sutures are tied to achieve hemostasis. No conclusions can be drawn with a small number of patients, but of the Impella and TandemHeart patients, bleeding complications tended to be less in the 15 patients with vs. the 11 patients without "preclosure", including TIMI minor bleeding (7% vs. 36%), TIMI major bleeding (40% vs. 64%), the incidence of (67% vs. 82%) and mean number of transfused packed red blood cells (3.9 vs. 6.9 units).

**Table 4:** Bleeding Complications

	Total	IABP	IMP	TH
TIMI Minor Bleed	22% (17/76)	24% (12/50)	14% (1/7)	21% (4/19)
TIMI Major Bleed	33% (25/76)	24% (12/50)	43% (3/7)	53% (10/19)
Bleeding Requiring Surgery	0% (0/76)	0% (0/50)	0% (0/7)	0% (0/19)
Retroperitoneal Bleed	0% (0/76)	0% (0/50)	0% (0/7)	0% (0/19)
Vascular Closure Device^a^	20% (15/76)	0% (0/50)	57% (4/7)	58% (11/19)
Other Reason for Bleeding^b^	22% (17/76)	20% (10/50)	14% (1/7)	32% (6/19)
Platelets (k/ul)				
Before	216 ± 85 (75)	231 ± 87 (49)	234 ± 51 (7)	171 ± 77 (19)
After	128 ± 65 (71)	137 ± 62 (47)	156 ± 73 (7)	91 ± 57 (17)
Before-After Change	-90 ± 71 (71)	-95 ± 65 (47)	-78 ± 90 (7)	-80 ± 83 (17)
Hemoglobin (g/dL)				
Before	12.4 ± 2.2 (74)	12.4 ± 2.1 (48)	11.9 ± 1.8 (7)	12.5 ± 2.7 (19)
After	9.7 ± 2.0 (71)	10.2 ± 1.5 (46)	8.5 ± 2.6 (7)	8.8 ± 2.4 (18)
Before-After Change	-2.7 ± 2.2 (71)	-2.3 ± 1.9 (46)	-3.4 ± 2.4 (7)	-3.7 ± 2.4 (18)
Transfusion				
Fresh Frozen Plasma	7% (5/76)	4% (2/50)	0% (0/7)	16% (3/19)
Platelets	9% (7/76)	6% (3/50)	0% (0/7)	21% (4/19)
Packed Red Blood Cells	47% (36/76)	34% (17/50)	57% (4/7)	79% (15/19)
Mean Number	4.1 ± 4.2	2.6 ± 1.2	4.5 ± 3.7	5.5 ± 5.9

^a^Perclose device (Abbott Vascular, Redwood City, CA) using the "preclose" technique; ^b^other reasons include coagulopathy, ventricular wall rupture, anemia at presentation, and pulmonary hemorrhage. IABP: Intra-Aortic Balloon Pump; IMP: Impella L.P. 2.5; TH: TandemHeart; CABG: Coronary Artery By-pass graphing; TIMI: Thrombolysis in Myocardial Infarction.

### Hemodynamics

**Figure 1:**
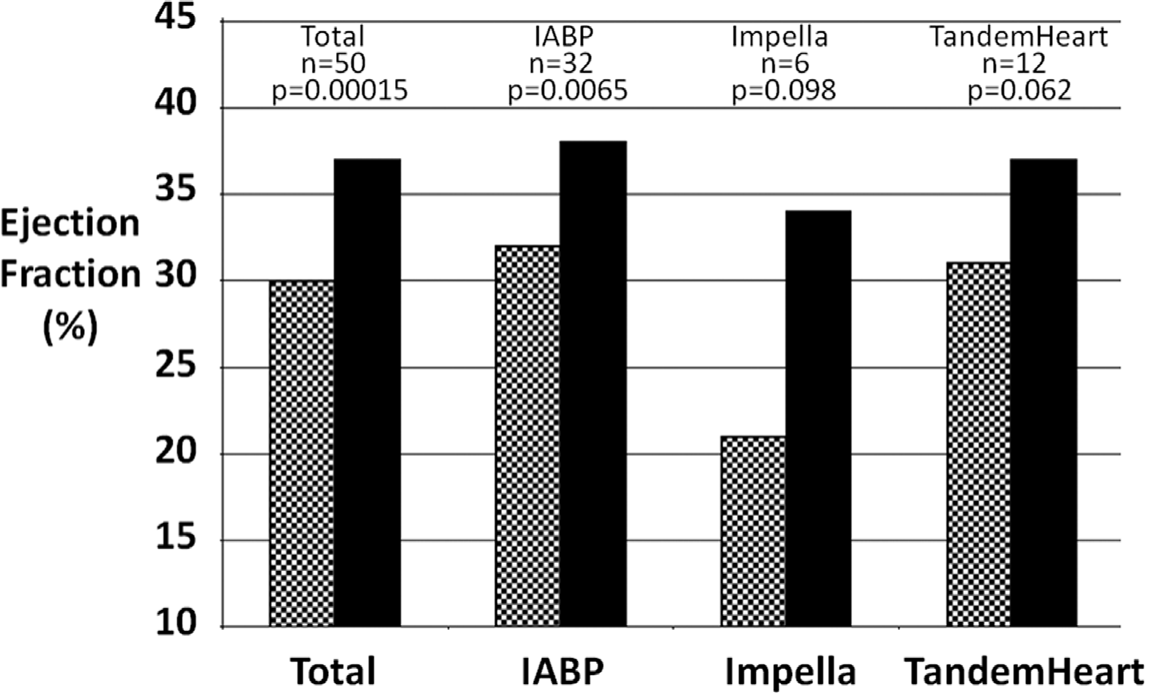
Change in ejection fraction before and after percutaneous left ventricular assist device support for cardiogenic shock. Mean ejection fraction for all patients with measurements before and after PLVAD support for cardiogenic shock. Dotted bar: before PLVAD insertion; Solid bar: after PLVAD removal; IABP: intra-aortic balloon pump.

Ejection fraction increased significantly from before to after device use in the overall cohort by a mean of 6.6±11.4% (P = 0.00015) as shown in Table 5 and the Figure 1. The increase in ejection fraction was significant for the IABP group (P = 0.0065) and tended towards significance in the Impella (P = 0.098) and TandemHeart (0.062) groups which were limited by a smaller "n". Furthermore, systolic blood pressure increased significantly in all patients and in the IABP group from before to after device use.

**Table 5:** Hemodynamics

	Total	IABP	IMP	TH
EF				
Before	30.4 ± 16.5% (62)	33 ± 16% (39)	22 ± 10% (6)	27 ± 18% (17)
After	38.8 ± 16.9% (60)	38 ± 14% (40)	34 ± 15% (6)	39 ± 21% (13)
Before-after change	+6.6 ± 11.4% (50)	+5.8 ± 11.3% (32)	+12.5 ± 15.1% (6)	+5.8 ± 9.7% (12)
Before-after P value	0.00015	0.0065	0.098	0.062
SBP^a^				
Before^b^	97 ± 23 (71)	97 ± 24 (48)	105 ± 25 (7)	93 ± 20 (16)
During	111 ± 21 (70)	115 ± 20 (48)	111 ± 26 (7)	101 ± 21 (15)
After	111 ± 16 (54)	114 ± 15 (36)	113 ± 16 (6)	98 ± 14 (12)
Before-during change	+14 ± 20 (70)	+18 ± 19 (48)	+6 ± 9 (7)	+6 ± 21 (15)
Before-after change	+12 ± 26 (54)	+17 ± 29 (36)	+4 ± 19 (6)	+4 ± 14 (12)
Before-after P value	0.00076	0.0015	0.621	0.307
MBP^a^				
Before	75 ± 18 (71)	76 ± 18 (48)	76 ± 18 (7)	73 ± 16 (16)
During	88 ± 18 (70)	89 ± 18 (48)	84 ± 21 (7)	87 ± 17 (15)
After	77 ± 11 (54)	77 ± 11 (36)	79 ± 11 (6)	78 ± 13 (12)
Before-during change	+13 ± 15 (70)	+14 ± 15 (48)	+8 ± 15 (7)	+13 ± 16 (15)
Before-after change	+1 ± 18 (54)	+1 ± 19 (36)	0 ± 22 (6)	+3 ± 14 (12)
HR				
Before	93 ± 23 (71)	95 ± 23 (48)	77 ± 17 (7)	93 ± 25 (16)
During	95 ± 21 (70)	95 ± 23 (48)	88 ± 15 (7)	98 ± 20 (15)
After	87 ± 16 (54)	86 ± 14 (36)	88 ± 22 (6)	90 ± 19 (12)
Before-during change	+3 ± 27 (70)	1 ± 28 (48)	+11 ± 28 (7)	+7 ± 25 (15)
Before-after change	-4 ± 25 (54)	-6 ± 25 (36)	+7 ± 24 (6)	-1 ± 25 (12)

IABP: Intra-Aortic Balloon Pump; IMP: Impella L.P. 2.5; TH: TandemHeart; RPM: Rotations Per Minute; EF: Ejection Fraction; SBP: Systolic Blood Pressure in mmHg; MBP: Mean Blood Pressure in mmHg; HR: Heart Rate; ^a^99% of the documented SBPs and MBPs were supported by vasopressors; ^b^in addition, 2 patients each with an IABP and TandemHeart had no detectable SBP.

## Discussion

This study indicates that in patients suffering from CS the Impella and TandemHeart devices can be initiated successfully and have major complication rates similar to patients treated with IABP alone, except for an increased incidence of major bleeding. A rapid improvement in ejection fraction suggests a favorable effect of PLVADs in conjunction with revascularization on the myocardium. Moreover, with the general approach of treating CS of escalating severity first with vasopressors and an IABP, reserving the Impella and TandemHeart devices for patients with the most severe hemodynamic compromise, the overall death rate compares favorably with previously published death rates of patients with CS.

### Survival

This was not a randomized trial and the groups differed hemodynamically prior to device insertion, so death rates are not comparable between groups within this cohort. The TandemHeart device was used in the patients with the most severe hemodynamic compromise including in 10 patients deemed IABP failures; 3 of these 10 patients survived. Likewise, the Impella device was used in 5 patients deemed IABP failures, 4 of whom survived. It is unknown how many of these 15 patients deemed IABP failures would have survived without Impella or TandemHeart support. The TandemHeart group had a higher mortality rate than the Impella group, but selection bias placed the patients with the most severe hemodynamic compromise in the TandemHeart group. This general approach of utilizing the IABP first, which can be initiated rapidly, and reserving the newer PLVADs for patients in whom the IABP was inadequate resulted in an overall mortality of 40%.

The most robust mortality data on patients with CS comes from the SHOCK trial [1] and registry [2, 3] (Table 6). The SHOCK trial included patients with STEMI complicated by CS due predominantly to left ventricular dysfunction and excluded patients with severe systemic illness, with a mechanical cause of shock, or if they were not suitable for revascularization. Ultimately, 1492 patients were screened, 302 were included in the randomized trial and the other 1190 were included in the registry. The 30-day mortality was 51% in the trial patients (86% of patients were treated with an IABP) [1]. In-hospital mortality was 60% in the registered patients [2, 3]. More recently, 3 randomized trials compared the IABP to either the Impella or TandemHeart devices [6-9]. Combining these 3 trials (total n = 100), 30-day mortality was 42% with an IABP and 45% with either an Impella or TandemHeart device (P = not significant). These trials, however, excluded patients with age > 75 years, sepsis, right heart failure, significant aortic regurgitation, mechanical complications of myocardial infarction, severe peripheral vascular disease and other comorbidities. Our trial did not exclude any patients and even included 8 patients who presented with cardiac arrest, 27 patients > 75 years, 9 patients with severe valvular disease, 4 patients with undetectable blood pressure and one patient each with ventricular wall rupture and papillary muscle rupture. Overall survival of 60% in this study compares favorably to these other reports with strict exclusion criteria, although each study is underpowered and no conclusions can be drawn about mortality rates. A trial that randomizes deemed IABP failures to either continued standard therapy or to an Impella or TandemHeart device seems warranted, although such a trial would encounter numerous challenges. Widespread use of the TandemHeart (∼$25,000) and Impella (∼$21,000) devices will probably be limited due to their cost until benefit is proven in large-scale randomized trials.

**Table 6:** In-Hospital Mortality Rates for Patients with Cardiogenic Shock

Report	N	Cohort	In-hospital Mortality
SHOCK trial [1]	302	STEMI with cardiogenic shock due predominantly to left ventricular dysfunction; excluded mechanical cause of shock, severe systemic illness, not suitable for revascularization	51%^a^
SHOCK registry [2]	1190	Patients excluded from SHOCK trial	60%^b^
Meta-analysis [7] of 3 randomized trials of IABP vs. Imp/TH	100	Excluded age >75, sepsis, right heart failure, aortic regurgitation, mechanical complications, severe peripheral vascular disease, other	42% IABP 45% Imp / TH
This study	76	All patients with percutaneous left ventricular assist devices; Excluded none	40%

^a^Mortality in the SHOCK trial was 47% in patients randomized to early revascularization and standard medical therapy and 56% in patients randomized to standard medical therapy (the difference was not significant at 30 days but reached significance at 6 months). ^b^Mortality in the SHOCK registry was 47% in patients treated with an IABP and thrombolytics, but baseline characteristics and revascularization rates favored this group.STEMI: ST-segment elevation myocardial infarction; Imp: Impella; TH: TandemHeart.

In this report, CPR was administered to 49% of the patients who had a mortality rate of 51%, which is very similar to the 48% of patients administered CPR with a mortality rate of 57% in a recent report [13] of 117 patients with refractory CS treated with a TandemHeart device (82% were treated initially with and deemed to have failed an IABP). Patients in their report more closely resemble our TandemHeart group, in which 37% received CPR with a mortality rate of 71%. The results of these two reports suggest that in patients with CS who are administered CPR, approximately half of those who are treated with an IABP survive, and of patients deemed IABP failures who are transitioned to a TandemHeart device an additional 30-45% survive. Furthermore, a retrospective analysis reported the outcomes of 10 patients treated with an Impella [6] or TandemHeart device [4] after not responding to vasopressors and IABP therapy [15]. Three (30%) of the 10 patients survived [15].

### Device success, angiographic success, and improved ejection fraction

All devices were initiated successfully, suggesting that device related complications are low with experienced operators in a private, community hospital setting. In this cohort of patients in CS, including many with STEMI and high risk coronary anatomy, angiographic and procedural success were very high, including 100% of the Impella and TandemHeart patients.

Interestingly, ejection fraction increased in each group from before to after device use, even though the final measurement generally occurred within 7 days of device discontinuation during which time the injured myocardiam is traditionally "stunned", or temporarily not functioning properly. Firm conclusions cannot be drawn from this retrospective study with a small sample size; however, two other small analyses reported improved ejection fractions in patients treated with an IABP (increased by 17%) and in patients treated with an Impella (increased by 8-9%) [8, 16]. Perhaps these devices have a beneficial effect on the myocardium that reduces myocardial stunning. In a canine model of myocardial infarction, compared with reperfusion alone, reperfusion during left ventricular unloading with the TandemHeart device significantly reduced infarct size and microvascular damage (P < 0.001 for each) [17]. Similarly, in an animal model of myocardial infarction with simulated bypass grafting, infarct size was reduced with an IABP and was further reduced with a TandemHeart-like device [18].

### Bleeding complications

This analysis confirms in "real-world" patients that bleeding is a major complication of PLVADs, as reported in previous trials [6-9]. There were no instances of retroperitoneal bleeding or bleeding that required surgery, indicating that blood loss was due primarily to hemolysis and bleeding from the access site. Vascular closure with the "preclose" technique appeared to reduce bleeding complications and should be considered if time allows. Transfusions of blood products should be anticipated when using PLVADs, especially the TandemHeart device.

### Major Complications

Major complications with the new PLVADs occurred infrequently and with a rate similar to the IABP. In contrast to the 3 randomized trials in which limb ischemia tended to occur more frequently in Impella and TandemHeart patients compared with IABP patients (relative risk 2.59, P = 0.13) [7], rates of limb ischemia were similar in our analysis (6% IABP, 8% combined Impella and TandemHeart). All instances of limb ischemia resolved upon device adjustment or removal.

### Limitations

As a retrospective analysis, data was limited to what was included in the medical record. As a study from a single center, it is unknown whether the results will translate to the interventional community. Sample size was small and device groups in this analysis are very different at baseline, which limits direct comparisons between groups and mortality conclusions. The blood pressure data was heavily influenced by concomitant vasopressor therapy which was continuously titrated to maintain adequate blood pressure in 99% of these patients. Additionally, many patients in the Impella and TandemHeart groups had IABPs in place before, during or after device use and patients who died with the device in place did not have hemodynamic measurements after device removal. Outcomes for most patients were only available in-hospital, not at 30 days. However, the SHOCK registry only reported in-hospital outcomes and in-hospital and 30-day mortality was similar in the SHOCK trial [3].

### Conclusion

In patients with CS, hemodynamic support with the Impella and TandemHeart devices can be initiated with a high degree of success and appear to have major complication rates similar to the IABP alone, except for an increased incidence of major bleeding. Randomized trials are warranted to investigate use of the Impella and TandemHeart devices initially for patients in CS (prior to IABP therapy). The general approach of treating patients in CS initially with vasopressors and IABPs, and reserving the Impella and TandemHeart devices for patients in whom standard therapy was inadequate resulted in an overall death rate of 40%. Randomized trials are warranted to investigate the Impella and TandemHeart devices in patients with CS refractory to IABP therapy.
